# Knowledge Families Hold: Co‐Production and Co‐Research With Mental Health Family Carers in Understanding Experiences During the COVID‐19 Pandemic

**DOI:** 10.1111/hex.70093

**Published:** 2024-11-06

**Authors:** Caroline Walters, Eileen McDonald, Carli Sheers, Kerry Hawkins, Hayley Solich, JulieAnne Anderson, Nevena Simic, Danielle Moore, Tony Stevenson, Sharon Lawn, Melinda Goodyear, Marcelo Maghidman, Melissa Petrakis

**Affiliations:** ^1^ Department of Social Work Monash University Melbourne Victoria Australia; ^2^ National Mental Health Consumer and Carer Forum Canberra Australian Capital Territory Australia; ^3^ Bipolar Australia Sydney New South Wales Australia; ^4^ Embrace Multicultural Mental Health Canberra Australian Capital Territory Australia; ^5^ Helping Minds Perth Western Australia Australia; ^6^ Mental Illness Fellowship of Australia Brisbane Queensland Australia; ^7^ Lived Experience Australia Adelaide South Australia Australia; ^8^ College of Medicine and Public Health Flinders University Adelaide South Australia Australia; ^9^ School of Rural Health Monash University Melbourne Victoria Australia; ^10^ Emerging Minds Adelaide South Australia Australia; ^11^ Mental Health Service, St. Vincent's Hospital Melbourne Victoria Australia

**Keywords:** caregivers, co‐design, COVID‐19, families, mental health

## Abstract

**Background:**

Through an in‐depth exploration of mental health family carers' experiences during the COVID‐19 pandemic in Australia, this co‐produced study identified recommendations for advocacy, practice and policy implications to uphold family carer wellbeing. Government‐enforced restrictions, changed service availability and difficulties accessing hospitals, led to additional anxiety, depression and elevated distress, especially for people experiencing mental health challenges before the pandemic. The National Mental Health Consumer and Carer Forum alongside two academic researchers aimed to discern the impact of care provision, levels of distress, unmet needs, challenges and benefits of providing support, across geographic locations and diverse communities.

**Methods:**

This article reports on the survey component of a co‐designed mixed‐methods exploratory study of family carer experiences. A project steering group worked with two academic researchers, members of SWITCH Research Group, Monash University, to develop the 71‐question online survey across 9‐domains.

**Results:**

Family carers were relied upon to provide support and care when mental health services changed or closed. Carers support more than one person and typically people with daily and high‐level needs. Caring levels increased from 26‐h to an average of 40‐h a week of support provision, with changed roles and increased complexity. Heightened demands became stress‐inducing to the point of mental ill health and suicidality for some family carers.

**Conclusion:**

Government policy and pandemic responses failed to address the financial, practical or emotional resources needed to fulfil the role of care provision and support to unwell and extremely distressed people with new or ongoing mental and psychological ill‐health.

**Patient or Public Contribution:**

From its inception, this project was co‐produced and co‐designed with mental health family carers and service users based on their expertise in understanding their experiences and ways to best explore these to the benefit and wellbeing of families in distress. The academic research partners both have active experience of supporting people with mental health challenges. Through each of the identified phases, lived experience expertise (family carers and service users) co‐designed and co‐facilitated the process. At times leading the process, such as in recruitment strategies, and at other times acting as guides. Guidance was provided by lived experience expertise in reflecting upon the literature review to understand what had been researched internationally and what would be important to understand in Australia. The academic partners advised on the possible processes for data collection, and the lived experience experts decided on the methodology based on that advice. Both the focus group and survey questions were developed and scrutinised from the perspective of the service users and carers in the project team. Difficult conversations were handled with respect, service users within the project team gently addressed areas of enquiry that may suggest stigma or feed into societal stereotypes of people with mental health challenges. Carers were able to consider the wording of questions to still be able to address areas of concern including domestic violence within the family unit and suicide. Dissemination strategies were planned together with the carer and service user representatives being co‐presenters at conferences. The report for submission to the National Mental Health Commission (Australia) was written and reviewed with all partners. A committee of service users and carers, alongside the academic partners, planned the launch of the report in August 2023. The co‐authorship of peer‐reviewed articles has included family carers and service users from the National Mental Health Consumer and Carer Forum.

## 1 Introduction

Recognising the ‘deep toll on the subjective wellbeing of carers’ [1, p. 621] in providing intensive support and care provision, this article reports on a co‐produced research project to understand the experiences of mental health family carers during COVID‐19 (the pandemic). During the pandemic, caregivers in general experienced increased stress, and studies noted higher distress in families of people who experience mental health challenges or psychological distress [2, 3, 4]. The provision of mental health services was impacted globally [5] with many services moved from in‐person appointments and inpatient provision to alternative service delivery modes, or ceased [2, 5]. Subsequently, family carers were required to fill the void in caring for family members who experienced mental health challenges and psychological distress.

A recent review [6] found a scarcity of studies focused on mental health family carers. Family carer studies indicated a diversity and intersectionality of the family and the service user's experiences, with growing needs for increased levels and complexity of support [7, 8]. For mental health family carers, the pandemic led to higher demands of informal support, with changed support roles and caring tasks. Additional caring roles, included undertaking physical checks, assisting with medications, responding to different health challenges, and adult children returning to live in parent's home for care and support [6, 9]. Few studies reported the experiences of family carers supporting people with mental health challenges during this intense period and even less research was designed and conducted alongside mental health service users and their families [10, 11, 12, 13].

During the first year of the pandemic, Australia experienced some of the highest Government‐enforced restrictions globally [14]. A key priority for the Australian Government was to monitor and model the mental health impact of the pandemic on vulnerable populations and understand requirements for mental health services and workforces [15]. The Australian Government acknowledged the pandemic could exacerbate or give rise to mental health conditions, and services might not be able to respond with appropriate medical and mental health care. The National Mental Health Commission [15, p. 9] noted:

In turn, this will have more significant impacts upon the mental health and wellbeing of carers who will need to support their loved one in a socially isolated household, and who may themselves be experiencing poorer mental health due to the restrictions and impacts from the pandemic.

The National Mental Health Consumer and Carer Forum (NMHCCF) was funded by the National Mental Health Commission to report on and make recommendations about the experiences of mental health family carers, an identified vulnerable population. The terms ‘family’, ‘family carer’, ‘informal carer’, ‘unpaid carer’ and ‘caregiver’ all appear in the literature, to represent people who are in a significant relationship with a person who lives with mental health challenges, mental illness or psychological distress and provides unpaid support and care. Family within this project encompassed all people in a significant relationship with a mental health service user, including family of origin (biological or nonbiological), or family of choice.

The funded NMHCCF project involved co‐designing two opportunities for family carer participation in research; through focus groups or an online survey. Co‐design is when people who are central to an issue, policy or problem become part of the design team, using their expertise gained through experience to improve the design [16]. This article reports on the methodology and findings from the survey. The findings from the focus groups are reported elsewhere [13].

### 1.1 Aim

Through research co‐designed in conjunction with family carers and mental health service users, the aim of this study was, to discern the experiences of mental health family caring; levels and types of distress, unmet needs, challenges and benefits of providing support, across geographic locations and diverse communities, during the context of the COVID‐19 pandemic in Australia.

## 2 Materials and Methods

### 2.1 Study Design

The commissioned research project was co‐produced with a Project Steering Group (PSG), with 23 members, composed of family carers and ‘consumers’ – a term indicating service users in Australia – representing the NMHCCF and organisations advocating for and supporting family carers across Australia. Co‐production is a form of project management that involves stakeholders and professional workers – in this case, university researchers – coming together to design, undertake and deliver a project [17]. The overall project co‐researched with family carers and service users to understand their experiences, especially in relation to the impact of the pandemic on caring responsibilities, unmet needs and levels of distress. The two academic researchers (C.W. and M.P.) have their own lived experiences of supporting people with mental health challenges. The project adopted a feminist‐informed approach to enquiry utilising co‐research to establish a dialectical process drawing on the ‘complementary perspectives, interests, skills and knowledge bases of academics and practitioners’ (mental health family carers) [18, p. 64]. Feminist methodologies recognise the process of research is as important as the outcome, with efforts taken to strengthen the connections between participants and researchers aiming to create environments which are caring, nonhierarchical and capacity building [19]. Reflexivity is important to providing insight and openness across phases of the research process creating opportunities for transformation [19].

**Figure 1:**
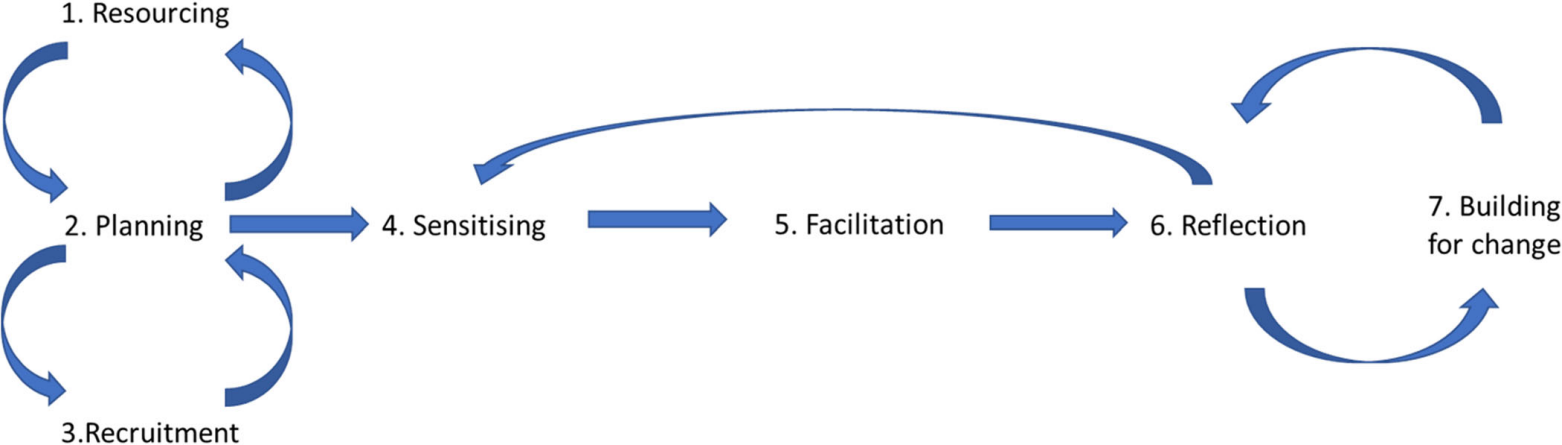
*A* co‐design framework. *Note*: Extended from J. Trischler, T. Dietrich and S. Rundle‐Thiele, “Co‐Design: From Expert‐ to User‐Driven Ideas in Public Service Design,” *Public Management Review* 21, no. 11 (2019): 1613.

A seven‐step framework (Figure 1), extending Trischler et al.'s [16] framework for co‐designing with vulnerable persons, guided the co‐production process. This framework recognises the iterative nature of co‐design work, building in reflection and sensing phases [16] and was selected as it has strong foundations in co‐design literature and aligns with feminist theories [19, 20].

The two academic researchers from SWITCH Research Group, Monash University and the PSG worked together over a 9‐month period from December 2021 to August 2022. The descriptive and exploratory mixed‐methods research was co‐designed through the 7‐step process including: (1) identifying resourcing; (2) planning engagement, recruitment and determining the research question and mixed‐methods approach; (3) recruitment – deciding processes for recruitment and ways to promote safety; (4) sensitising through reviewing the literature; (5) facilitation through co‐designing the national survey instrument, and supporting data collection; (6) reflection through analysing and interpreting results and (7) building for change through preparing the final report, identifying recommendations and participating in the dissemination strategy. The co‐created research question was: What were the experiences, quality of life and wellbeing of family carers of people with mental health challenges, mental illness or psychological distress, including within diverse geographical locations and communities, in relation to their caring responsibilities and needs during the COVID‐19 pandemic in Australia?

The mixed‐methods approach, noted as valuable in social research for providing a more holistic investigation with complex topics [21], incorporated fixed‐ and open‐ended responses within the survey, alongside the focus groups reported elsewhere [13]. The convergent parallel design enabled a broader and deeper understanding of the experiences of family carers, quantified the impacts of the pandemic, elaborated on reasons for the impact and proposed suggestions for resolution [21].

**Figure 2:**
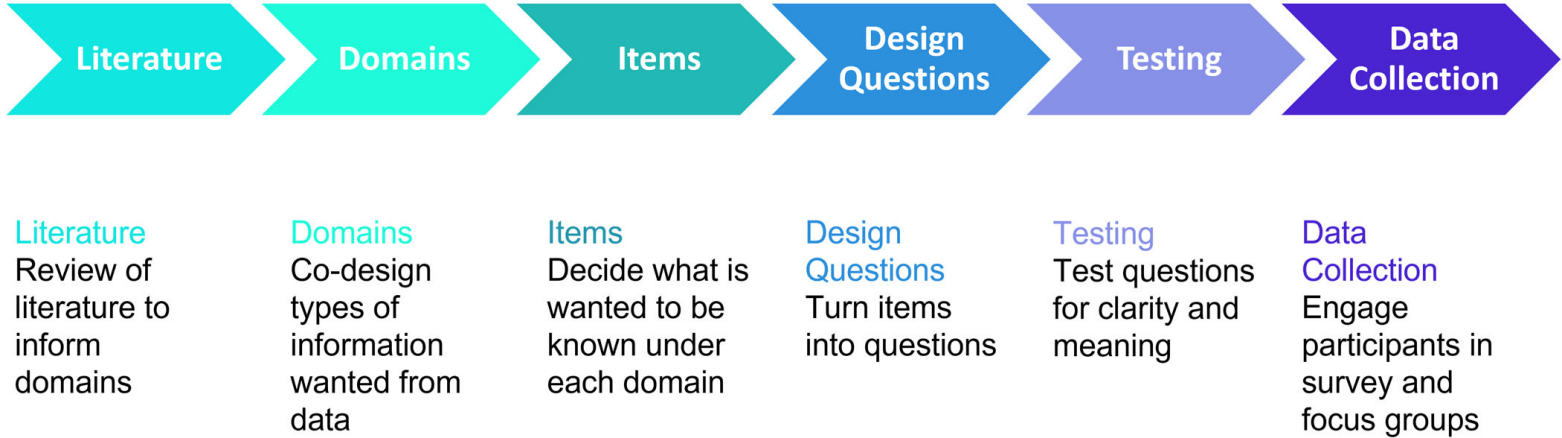
*Process of survey development*.

The academic researchers worked with the PSG to prepare the survey, through a stepped process (Figure 2), across three 2‐hourly meetings.

The group decided upon nine domains of questions with a total of 71 questions (Table 1). The domains, items and questions were carefully considered in relation to the literature (current knowledge), and their purpose in revealing experiences that were known to the PSG but were not often visible within research evidence or policies. Some members expressed concern at the length of the survey, however, due to limited peer‐reviewed research and opportunities to gather information about family carer experiences, the group reached a consensus on the number and format of questions.

**Table 1:** Domains and items of the questionnaire.

Domain of questions	Items	Number of questions
Demographics	Geographic region, age, gender, cultural or group identity, language spoken, education level	9
Who did family support?	Service user cultural or group identity, relationship, number of people supported, mental health challenges of supported person, people supported with other challenges	5
Level of support	Length of caring role, living arrangements, number of people in the household, hours of support and changes	7
Where support provided	Mode of support, where support provided, changes and impacts	4
Pandemic effects on caring and stress	Level of stress resulting from pandemic responses, changing stress levels, amount of support needed for activities, distress level prior to before and during the pandemic, challenges, coping strategies	9
Learnings from the pandemic	Learnings for self and family members, greatest joy	2
Services during the pandemic	Experiences of supported person and carer, experiences with NDIS, impact of changes on family carer, what has helped, what would like to happen, other services	7
Family carer wellbeing	Personal health and wellbeing, thoughts of suicide, access to support	4
Domestic violence	Experiences of feeling unsafe or controlled, seeking assistance and help	6
*Family carer's own experiences of services*	*Help seeking behaviour,*	*3*
*Employment, education, social and economic impacts*	*Changes in hours of work, employment change, education activity changes, government support, financial resources and supporting others, social support and connection, seeking social, legal and economic support services and how helped*	*13*
*Future vision*	*Changes or innovations that would help*	*2*
	Total number of questions	71

*Note:* Italicised items are not reported here but will be explored in a subsequent paper.

The current study aims to report on the first 47 of 71 questions, introducing demographics and family carers' experiences, views about services and impacts on wellbeing. The remaining questions will be addressed in a paper to be written from a human rights framing, addressing family carer rights to be part of society and have security in relation to employment, connections and safety. The PSG collectively reviewed each question, the placement in the survey, alongside prompts for the participant to take a break or seek help if needed. The PSG suggested advising participants they could navigate back and forwards, and also take breaks and return to the survey.

Depending on the level of detail provided, it was estimated completing the survey would take between 30 and 45 min. Resources identifying support organisations were placed throughout the survey as well as provided in a downloadable document. The survey was created on the Qualtrics [22] online platform and all members of the PSG were able to pilot test the survey online, to check the ease of understanding questions and flow, with final changes made before approval for distribution.

### 2.2 Participant Recruitment

The online survey was available from the 9 May 2022 until the 18 June 2022. To support people completing the survey, if needed, a direct link was distributed to a purposive sample of mental health family carers via targeted emails or in newsletters of affiliated organisations of the NMHCCF. Family, friends, carers or supporters of a person with mental health challenges, mental illness and/or psychological distress, who were 18 years or older, were invited to participate. Participants were screened with a beginning question: Are you a family member, friend, carer, or supporter of a person with mental health conditions or challenges, mental illness or psychological distress? People who responded no, were thanked for their time and provided with sources of information for all carers.

### 2.3 Ethical Considerations

The Monash University Human Research Ethics Committee was satisfied the study met the requirements for the National Statement on Ethical Conduct in Human Research and granted approval (Project Id 31516). The front page of the survey had a link to the explanatory statement and people were advised participation in the research was voluntary. Completion of the survey indicated consent to participate.

### 2.4 Data Analysis

For set‐response quantitative questions, exploratory data analysis was undertaken using descriptive statistics to summarise the responses, including frequency distributions (number, percentage), measures of central tendency (mean, standard deviation) and variability (range, standard deviation). One sample *t*‐test was used to determine the difference between hours of providing support and stress levels before and during the pandemic, at a significance level of *p* < 0.05.

The first author (C.W.), an experienced social work researcher, with mental health family carer lived experience, completed the initial qualitative analysis of the open‐ended response questions, utilising reflexive thematic analysis from an inductive experiential realist framing [23] to identify patterns of meaning and explanation in the open‐ended question responses. The downloaded extended responses and initial findings were discussed with the senior author (M.P.) before being presented and reflected upon in a meeting with the PSG. Members of the team considered how these responses were similar to those of the focus groups [13], within the literature [6], their own experiences or whether they provided different experiences and meaning. Some key differences to the literature in the survey analysis were findings specific to the Australian context. Members of the team themselves noted the challenges of providing support across state jurisdictions and within enforced restrictions.

## 3 Results

Themes (Table 2) from the first 47 questions, reflected those in the literature [6], being diversity and intersectionality of mental health family carers in the demographics, similar reporting of the experiences of family carers in providing support, and further elaboration on the impact on the quality of life and wellbeing of family carers.

**Table 2:** Themes evident within survey responses.

Theme	Sub‐theme
Experiences of family carers in providing support	Inadequate service and system response
	Increased demand for support from family carers
	Increased complexity and variety in types of support
Impact on family carer quality of life and wellbeing	Impact on levels of stress experienced
	Impact on family carer wellbeing
	Impact on family unit distress
	Impact on family carer help seeking
	Thoughts of suicide in family carers

### 3.1 Participant Demographics

Upon closure of the survey on the 18 June 2022: 190 people commenced the survey; 11 people answered no to the screening question and were exited from the survey; and 101 completed the survey. All states within Australia, except the Northern Territory, were represented; Australian Capital Territory (2), New South Wales (32), Queensland (11), South Australia (4), Tasmania (3), Victoria (38) and Western Australia (11). The majority of people (70, 69.3%) identified as living in Metropolitan areas, 26 regional and 4 rural. Most participants (97, 96.0%) spoke English as their first language, with 2 people speaking Hindi, 1 Filipino and 1 Greek. No‐one reported they needed assistance with completing the survey. The majority (88, 87.1%) of the participants identified as female, with only 8 males, 3 who identified as nonbinary, and 1 preferring not to state. Ages ranged from 18 to 80 years, with the average age being 54 years, and the majority being between 40 and 60 years (48), 37 were aged between 61 and 80 and 14 between 18 and 39 years.

All participants had completed senior level of schooling, with 30 people completing vocational training, 22 an undergraduate degree, and 43 a post‐graduate degree. The majority of the participants were long term providers of support and care, with many having intersectional identities, including as a service user (37). Many family carers were working within mental health services as either a clinicians (14) or lived experience workers (31).

### 3.2 Experiences of Family Carers in Providing Support

#### 3.2.1 Inadequate Service and System Responses

Services were changed, disrupted or discontinued due to the pandemic (Table 3). Changes to services reported included being unable to pay service fees, being difficult to source, service users not wanting to engage, and not knowing if services were being used as the family carer wasn't in contact with the person at that time.

**Table 3:** People's Experiences of Services During the Pandemic.

How have the people you supported experienced services since the beginning of the COVID‐19 pandemic?		
	Number	Percent
They have lost services	33	32.7
Their services changed	50	49.5
They have gained services	12	11.9
They have found new services	18	17.8
This is their first episode of mental health challenges	14	13.9
Their services moved to online	49	48.5
Their services moved to via the phone	49	48.5

When asked in an extended response question about their greatest challenges during the pandemic, many family carers reported on the experiences with services. Poor experiences included not being able to visit the supported person in hospital, the person being discharged whilst still unwell (including active thoughts of suicide), extended waiting lists, not being able to access services the supported person felt comfortable using (often reported as wanting in person meetings), services no longer being available when a person moved location, lack of carer supports and inadequate information from Services and Government representatives about rules and how to provide care. One family carer wrote ‘…needing face to face support and not being able to get it has been difficult. Online support is okay but is not the same’ (ID74).

When asked open‐ended questions about their experiences with services, family carers identified feeling excluded. Family carers reported providing high levels of mental health care during the pandemic, and yet were not able to access services for supported people in need and not being included in planning and decision‐making about treatment they were then expected to monitor or enable. Many reported accessing information from the internet, but also identified this took time. Other sources of information included published reading material, friends, online applications and supported family carer activities through hospital services. A participant who provided support for an aging relative alongside a person with mental health challenges noted, ‘I was told I could get support as a carer for my father who is aging but not my partner as caring for someone with mental health is not acknowledged’ (ID122).

Participants had mixed experiences with the move to remote service provision. Whilst some family carers identified benefits of online and phone health services, including for themselves, such as reduced costs and less time in attending, many identified that the lack of in‐person engagement with service providers led to the person they supported disengaging from the service, ‘my daughter hated online video therapy…attended less often as a result’ (ID113).

#### 3.2.2 Increased Demand for Support from Family Carers

Family carers reported providing increased levels of support including significantly higher hours of support (*M* = 40.35, SD = 43.94) within the last 2 weeks during the pandemic, than hours of support (*M* = 26.30, SD = 33.07) before the pandemic, *t*(95) = −5.644, *p* < 0.001. There was a small to medium effect size (Cohen's *d *= 0.36).

The majority indicated caring for more than one family member with mental health challenges; with 51.1% supporting two or more people with mental health challenges (Table 4). Most tended to be long‐term carers with 75% reporting over 6 years of support, while 14% providing less than 2 years of support.

**Table 4:** Relationships of supported people.

How many and what is the relationship of people with mental health challenges being supported?				
	1 person	2 people	3 people	4 or more people
Under 16 years	8	4	3	1
Young person (16–25 years)	31	6	2	3
Adult child (over 25 years)	31	7	2	3
Partner	33			
Parent	17			
Sibling	11	2		
Another relative	6	2		
Friend	6	8		3
Work colleague	5	1		
Ex‐partner	2			

The majority of participants were living with the person they supported both during and before the pandemic (59.4%). For 11.9%, the supported person moved in with them during the pandemic, and a further 6.9% had the person occasionally live with them. Other arrangements included people living alone independently (33.7%), with another relative or friend (20.8%), in extended hospital stay (4.0%) or supported accommodation (6.9%). The majority (57.4%) of family carers reported a change in the way they provided support, with 73.3% providing support in the family home, almost half (47.5%) in person outside the family home, 29.7% provided support online and 58.4% via the telephone.

Open‐ended responses indicated the greater hours and need for support had an impact on family carers with less time for their needs. One participant noted; “Increased vigilance and sense of responsibility for their [supported person] wellbeing. Increased stress and anxiety for [my]self. Less time for my needs”. (ID24)

The reported less time for themselves sat alongside increased tensions within the family unit, with one participant writing:

Challenging and tough to hear the depths of depression, self‐harm and suicidal ideation. Challenging to have conflict with partner around how to best support our child. Intensity of family relationships during periods of isolation, with the mix of introverts and extroverts, and the different responses to online learning, working and isolation was tough. (ID10)

#### 3.2.3 Increased Complexity and Variety in Types of Support

**Figure 3:**
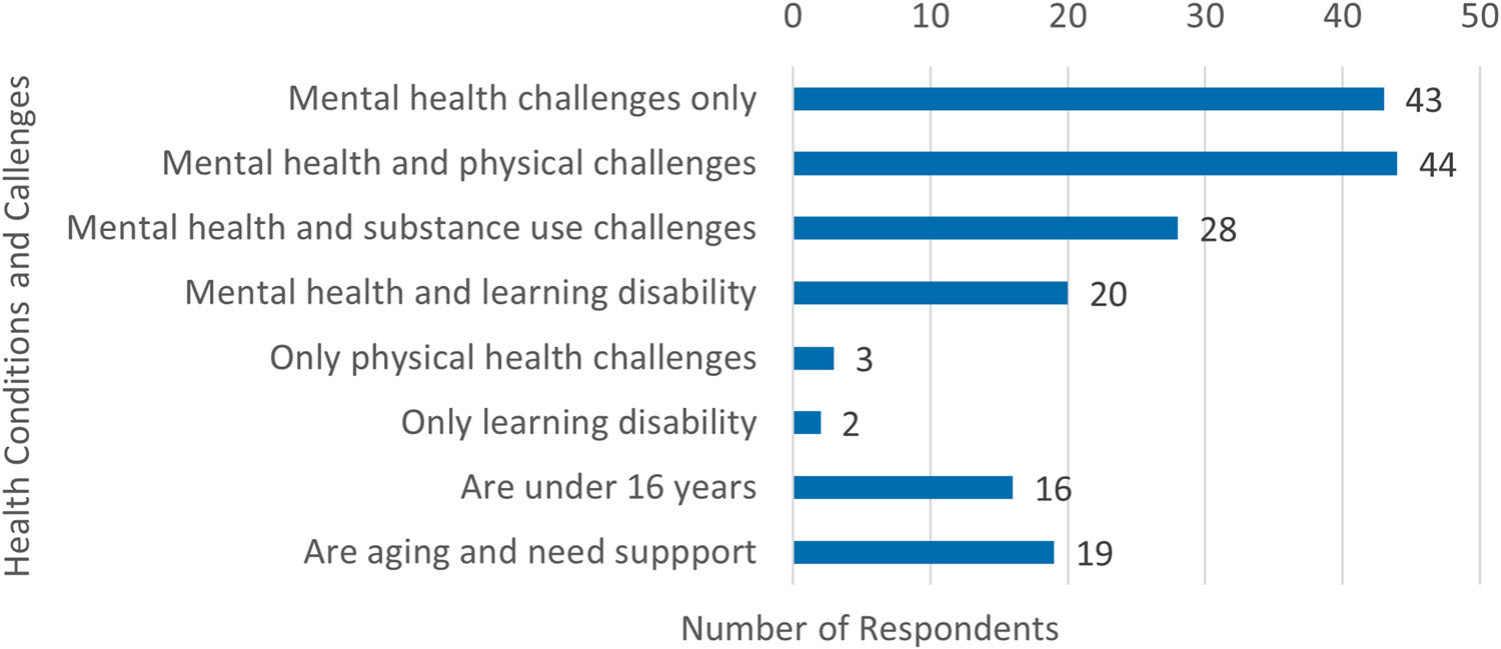
*Diverse care and support needs*.

Family carers provided support to people with diverse identities including multicultural (14), LGBTQI+ (16) and 15 people identified by the family carer as service users were also a caregiver. Some service users were part of the mental health workforce with four being clinicians and 14 lived experience workers. Many family carers (42.5%) provided support to people with multiple challenges, including other health challenges, who were aging or children (Figure 3).

**Figure 4:**
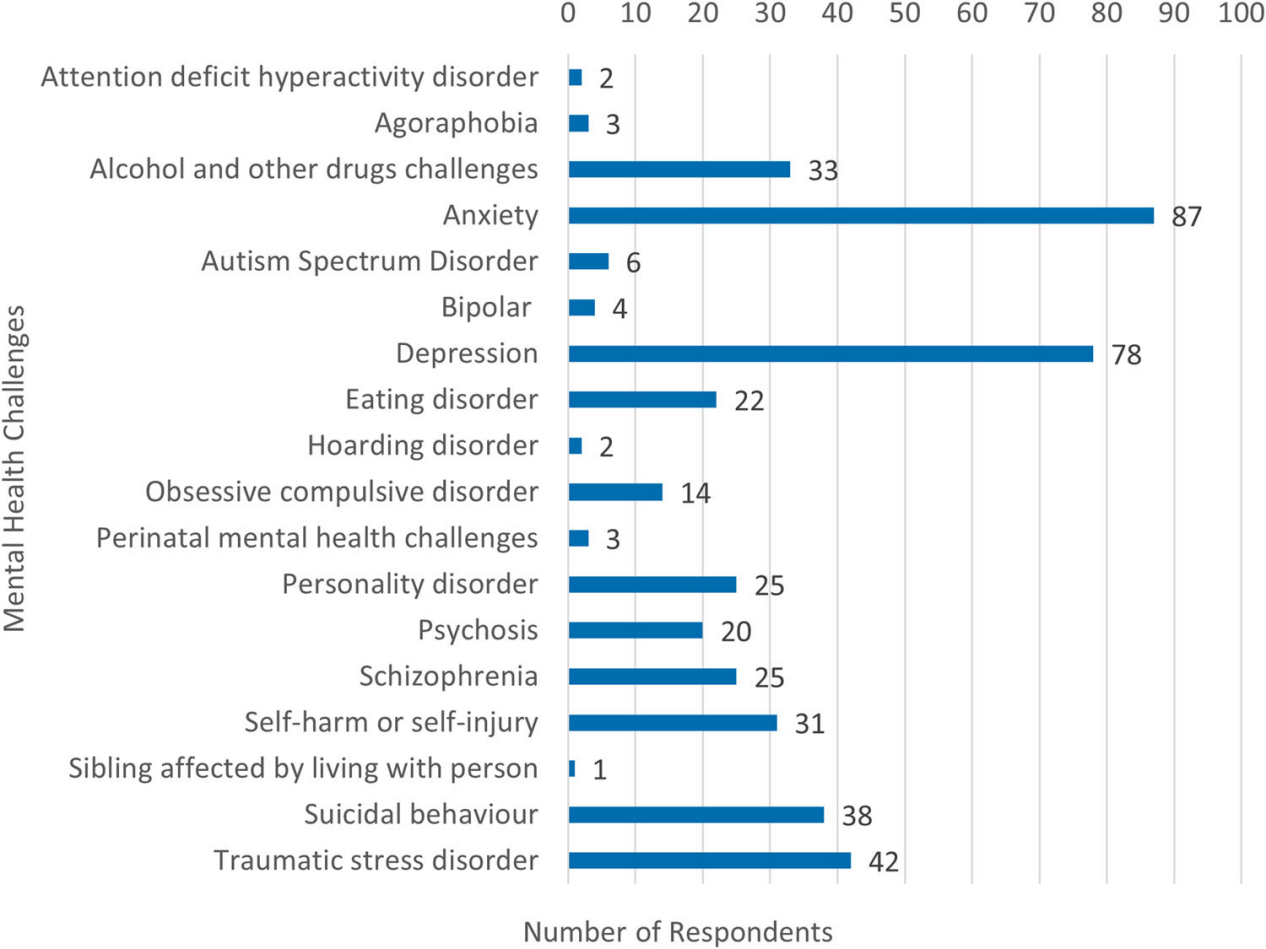
*Mental health challenges of supported person*.

Family carers also provided support to people who experienced a diversity of mental health challenges (Figure 4).

Of note, 49 participants were supporting someone who was experiencing either suicidal or self‐harm behaviours, with 20 family carers supporting people experiencing both self‐harming and suicidal behaviours. The experiences of these family carers were often one of being alert and vigilant 24‐h‐a‐day, 7‐days‐a‐week; ‘When my child disengaged from their case manager, I felt like I was the only one engaging in their life’ (ID10), ‘No chance to catch my breath, as I was covering for inadequacies in service delivery’ (ID112) and ‘Seeing your child in crisis with no access to mental health support was devastating to the whole family. We were all broken and unable to support each other’ (ID23).

Family carers also experienced complexities and stress around the impacts of the pandemic on the support they provided (Table 5), including being worried about becoming unwell, receiving support from others and increased responsibilities.

**Table 5:** Stress of pandemic‐related impacts.

How stressful were the following caregiving experiences compared to before the pandemic?	More	The same	I did not have this experience
I worry about getting sick and not being able to provide support and care	60.4%	22.8%	7.9%
Opportunities are limited to receive help from other care provision sources (such as other family members, community organisations, or home support services)	60.4%	14.9%	11.9%
Spending more time with the person I support	58.4%	22.8%	8.9%
Having other responsibilities, such as paid work, running a business, caring for children	57.4%	22.8%	15.8%
Limitations to being present with the person I support while they are in the hospital or during healthcare appointments	54.5%	16.8%	24.8%
Connecting with new supports	50.5%	19.8%	21.8%
My ability to visit the person I support is limited	48.5%	16.8%	23.8%
Getting information from the person I support's healthcare providers	40.6%	34.7%	18.8%
Accessing the medications and medical supplies that the person I support needs	36.6%	36.6%	20.8%

Restrictions arising from the pandemic and loss of services meant significant changes to the activities of support and also within the life of the family carers (Table 6). Most participants reported increased provision of emotional, practical, nutritional and financial support.

**Table 6:** Activities of support during COVID‐19.

Activities and level of support and care provided compared to before the pandemic	More	Same	Less	Not needed
Providing emotional support	71.3%	17.8%	5.0%	3.0%
Providing companionship and time together	62.4%	18.8%	11.9%	4.0%
Encouraging or helping with being active	49.5%	30.7%	7.9%	8.9%
Supporting leisure activities	48.5%	25.7%	10.9%	10.9%
Monitoring physical status	47.5%	28.7%	4.0%	14.9%
Shopping for groceries and essential items	46.5%	31.7%	6.9%	11.9%
Coordinating services	45.5%	27.7%	3.0%	19.8%
Providing nutritional support	44.6%	31.7%	2.0%	18.8%
Supporting effective sleep practices	44.6%	26.7%	3.0%	21.8%
Providing financial support with your money	43.6%	26.7%	3.0%	23.8%
Scheduling healthcare appointments	39.6%	30.7%	5.0%	17.8%
Contacting providers of services	38.6%	35.6%	4.0%	18.8%
Assisting with activities of daily living (assistance with personal care routines, making meals, etc.)	38.6%	28.7%	5.9%	21.8%
Managing suicidal thoughts and behaviours	37.6%	23.8%	3.0%	31.7%
Reminding about medications	32.7%	39.6%	3.0%	19.8%
Monitoring self‐injury or self‐harm	31.7%	19.8%	2.0%	42.6%
Assisting the person with managing their money	26.7%	33.7%	3.0%	33.7%
Monitoring harm with alcohol intake	24.8%	16.8%	5.0%	49.5%
Assisting with applying for NDIS package	21.8%	8.9%	2.0%	61.4%
Monitoring harm with drug or other substance use	18.8%	14.9%	4.0%	56.4%
Managing NDIS package	17.8%	13.9%	3.0%	58.4%

### 3.3 Impact on Family Carer Quality of Life and Wellbeing

Open‐ended responses indicated the impact of this increased level and complexity of support provision by carers led to increased demands on time, emotions and finances. Families reflected on their experiences:

I have missed a lot of work and don't have much sick leave or long service [leave] left. I need to care for her on my own without other support and this full‐time responsibility is challenging. I feel so alone in this. (ID106)

The following attests to the adverse impact of policies and planning that failed to address the needs of family carers who were left to hold and provide support to extremely unwell people with mental health challenges – including adverse effects on their physical and mental wellbeing and substance use:

Exacerbation of major depression, marked concern for all family members, feeling helpless and hopeless as I could not provide the level of care to family and friends that I previously supported, the weighty burden of isolation, my home was formally my sanctuary but work‐from‐home saw it transformed into a highly stressful prison, breakdown in workplace communication and accountability as the entire organisation moved to work from home which was hugely stressful and frustrating, worried about friends/family becoming unwell due to COVID, lack of access to health care services, inability to engage in social activities and travel (even local) became oppressive, poor sleep patterns, lack of motivation, increased alcohol use, weight gain and poor nutrition. (ID56)

#### 3.3.1 Impact on Levels of Stress Experienced

A one‐sample *t*‐test explored the difference between levels of identified distress of family carers before the pandemic and within the past 2 weeks of completing the survey. Family carers reported significantly higher levels of stress within the past 2 weeks of completing the survey (*M *= 6.34; SD = 2.442) than before the pandemic (*M *= 4.83; SD = 2.108), *t*(99) = −6.244, *p* <0.001. There was a medium to large effect size (Cohen's *d *= 0.66).

Participants were asked to rank the time during the pandemic when they experienced most stress. The beginning of the pandemic was ranked as most stressful (30.7%), with the period during the survey (June 2022) ranked by 16.8% as most stressful. The period of January to June 2021 was ranked by the least number of participants (11.9%). Open‐ended responses identified factors leading to the rankings at the beginning of the pandemic being: the initial uncertainty and changing rules leading to stressors of how to provide support within the restrictions, especially if the person was living away from home; increased distress of the people they supported, including suicidal behaviours; as well as the difficulty of supporting first episodes of psychological distress. Participants also identified the lack of respite available for people who were providing support in their own home. Towards the end of the pandemic, the loss of Government financial protections led to uncertainty including people being evicted from their rental properties, loss of income support and insufficient income to cover the basics. During this period, the reduction in mandated health protective measures led to an increased incidence of COVID‐19 viral infections in family members. The ongoing nature of the pandemic adversely impacted mental health challenges creating breakdowns in family units. A mother supporting two young people (under 25) and partner, who were experiencing anxiety, depression, suicidal thoughts and self‐harming behaviours described her experience as:

This has impacted my job, my relationships, my mental health, my ability to care for myself and my other children, my ability to contribute to the care of my elderly parents; my ability to contribute to society. Everything has been impacted negatively. (ID114)

To gain further understanding about the influence of the pandemic, participants were specifically asked to identify the level of stress occurring through COVID‐19 related stressors (Table 7). Most of the impact related to concerns around people becoming infected with the virus, alongside maintaining lockdown restrictions.

**Table 7:** Stress experienced by families.

Degrees of stress experienced by family (scale 1 not stressed to 6 extremely stressed)		
	Mean	SD
Concerns about the people you support becoming infected with COVID‐19	4.51	1.40
Another member of your family or close network being infected with COVID‐19	4.35	1.36
Concerns about yourself becoming infected with COVID‐19	4.28	1.34
Maintaining lockdown restrictions	4.20	1.25
A member of your family or close network dying from COVID‐19	3.86	1.78
Accessing testing facilities	3.42	1.59
Accessing accurate information about COVD‐19	3.28	1.39
Assisting people you support to get vaccinated against COVID‐19	3.24	1.64

#### 3.3.2 Impact on Family Carer Wellbeing

Family carer participants were asked to rate their own wellbeing (Table 8). The majority (59.4%) felt their overall wellbeing was poor to fair. Other areas rated as poor to fair included energy levels, exercise activities and sleep practices.

**Table 8:** Family carer's personal wellbeing.

How would you rate your personal health and wellbeing?					
	Poor	Fair	Good	Very good	Excellent
Overall wellbeing	18.8%	40.6%	21.8%	15.8%	0.0%
Energy levels	40.6%	24.8%	26.7%	5.0%	0.0%
Exercise activities	40.6%	24.8%	22.8%	7.9%	1.0%
Sleep practices	37.6%	28.7%	22.8%	6.9%	1.0%
Physical health	29.7%	25.7%	30.7%	10.9%	0.0%
Hopefulness for the future	25.7%	25.7%	23.8%	17.8%	4.0%
Mental health	24.8%	38.6%	20.8%	11.9%	1.0%
Balanced eating	24.8%	25.7%	31.7%	12.9%	1.0%
Ability to achieve things that are important to you	23.8%	33.7%	24.8%	11.9%	3.0%
Quality of life	16.8%	36.6%	29.7%	10.9%	3.0%
Management of substance use	5.0%	8.9%	18.8%	11.9%	42.6%

In open‐ended responses, family carers expressed some of the personal health and social challenges as long‐term concerns that existed before the pandemic. ‘I have felt isolated in my caring role for the larger part of thirty years until early this year when I started to participate in the Uniting Family and Carer support program’ (ID109). Similarly, another participant identified the long‐term adverse effects of poor system engagement with carers, ‘A strong word. But the isolation of being blamed for my daughter's mental illness is deeply distressing. Fortunately, I have supportive family and friends’ (ID71). The support from people who have been in similar situations such as peer workers or peer support groups were identified as helpful in reducing the sense of isolation and being invisible.

#### 3.3.3 Impact on Family Unit Distress

Family carers spoke of the trauma and of a sense of outsider witnessing, where seeing a child, or family member in crisis with no access to mental health support was devastating to the whole family.

We were all broken and unable to support each other. Mental health and wellness have since become a priority, as such the costs associated (enough to hit our Medicare threshold by April this year) required both adults to take up full time work. (ID23)

The impact on both the service user and the family was reported as distressing when long‐term hospitalisation occurred without contact, especially on young children:

Inpatient admissions were very distressing for my husband and for our family. He was isolated from his supports during admissions due to risk to the community being on hospital grounds…the nature of his admission [meant] he was away for 6 weeks with nil face to face contact with his children who are all under 11 years. This was their first experience of not being able to see their Dad when he was sick. This was a very difficult time for all of us and the transition back home was overwhelming for my husband. (ID52)

Family carers reported that as a result of the pandemic they were unable to receive practical (64.4%) or emotional (58.2%) support from family and friends when they needed it.

#### 3.3.4 Impact on Family Carer Help Seeking

Many family carers identified seeking support for their own needs through psychological services and counselling, whilst others reported not accessing services due to the cost, lengthy wait times, or no service availability. The general practitioner was the most utilised support service (54.5%), followed by online or telephone family support services (36.6%), private health services (28.7%), online counselling (21.8%), in‐person counselling (15.8%), mental health community service (10.9%) and in‐person family support services (9.9%). Family carers reported they prioritised services for the person they supported (28.7%) and couldn't afford services for themselves (23.8%). Family carers also utilised support groups (28.7%), away‐from‐home respite (10.9%) and in‐home respite (5.9%). Family carers (77.3%) identified they were unable to participate in social activities and 59.4% rated their family life in general as fair to poor. Participants hoped a future system would provide respite that recognised both the need of the family carer and the value to the person they supported. It was noted the importance of respite for family carers to have time to think about and prioritise needs for the supported person and their own health needs. ‘Regular flexible respite options for myself as a carer based on my needs, that will also provide the right kind of care for those I care for so I can take breaks’ (ID90).

#### 3.3.5 Thoughts of Suicide in Family Carers

More than 1 in 4 carer participants reported they had thoughts of suicide during the pandemic (Table 9). The 27 family carers who reported thoughts of suicide were asked if they were able to access help, with the majority either not receiving help or not seeking support.

**Table 9:** Family carer thoughts of suicide.

	Yes (*n*, %)	No (*n*, %)	Missing (n, %)
During the pandemic, have you had thoughts of suicide?	27, 26.7%	73, 72.3%	1, 1.0%

	**Yes (*n*, %)**	**No (n, %)**	**I did not seek support (n, %)**
During the pandemic, were you able to access support for your thoughts of suicide?	11, 40.7%	5, 18.5%	11, 40.7%

In responding to open‐ended questions about thoughts of suicide, family carers wrote this was in the context of excessive demands in relation to work and caring responsibilities, a sense of isolation and feeling helpless and powerless to help the supported person. Being in close contact with a loved one who was distressed, threatening or themselves suicidal, housing instability and relationship breakdowns left people feeling exhausted and unsure how to carry on. Those identifying as young carers spoke of isolation from friends and lower amounts of support from peers and academic professionals resulted in a lack of energy, motivation and interest in living.

When asked about the circumstances around the thoughts of suicide, family carers identified it was a way of escaping the difficulties of coping, supporting someone with suicidal behaviours, of being overwhelmed, especially around managing ongoing responsibilities for people's wellbeing *–* ‘My son almost lost his life 5 times, I didn't think I could cope any longer. But I did and I never had any intention of following through’ (ID9). Family carer's role in providing support for someone, in many cases being the only person left in the supported person's life, was reported as stopping family carers from acting on their thoughts of suicide.

## 4 Discussion

A co‐designed convergent parallel mixed‐methods descriptive and exploratory approach obtained a fuller picture of the multiplicity of roles and experiences mental health family carers had during the pandemic. Of note was the high response rate of females within the study, which may reflect the gendered nature of caring [24, 25, 26], or more importantly, the gender dynamics that occur within pandemics with the ‘downloading of care responsibilities onto women’ [27, p. 362], where women absorb the greater burden of care [28]. Before the pandemic, there was growing evidence of mental health systems relying on family carers to step beyond their familial roles into providing therapeutic and safety measures [12, 29]. Findings within this study indicated inadequate service and system responses to the pandemic leading to increased service users' need for informal support; increased complexity and variety of the types of support required; and a corresponding economic, psychological, social and health adversity for family carers.

Family carers reported higher levels of stress at different parts of the pandemic due to many factors. People who were already experiencing mental health challenges before the pandemic had additional anxiety and depression brought about by fears around the virus, restrictions to movement, alongside reduced and changed service availability, structure and access to hospitals. Similar to international studies, these loss of services, changes in candidacy to receive services and the format of service provision – largely moving to remote provision – impacted the way and where care was provided leading to increased demands on family carers in both the hours of caring but also in the emotional and practical aspects of providing support [5, 6].

Changes in work and living arrangements and state‐enforced restrictions enacted to combat the spread of the virus added complexities to family support provision. Family carers managed increasingly complex systems for navigation, growing advocacy needs and supporting and de‐escalating extreme distress. Assistance with navigation of services was reported as highly valuable to family carers and service users, including the value of receiving this from a carer or service user peer worker. Similarly, support with navigation alongside continuity of care through a known service provider or navigator has been recognised across studies [12, 30].

As restrictions continued, participants especially noted that young people were experiencing crises and that support were not available, or service users were being placed on long waiting lists for services. This extended wait list time included when people were experiencing thoughts of suicide resulting in the family carer being sleep‐deprived due to providing constant observation. This period marked a rise of eating disorders in young people, with similar findings pointing to an increased need for practical and monitoring support [31]. Internationally, studies have shown the challenges for family carers in supporting a person with suicidal or self‐harm behaviours [32, 33].

The impact of the pandemic and changes to support provision led to a decreased overall wellbeing of people in this study including reported reduction in mental and physical health, and poor sleep practices. Carers spoke of severe and persistent fatigue, of being overwhelmed and ‘burnt‐out’, reducing their capacity to provide support to all family members. Within this study, it was reported by 1 in 4 carers, the emotional intensity and heightened levels of demands and responsibility became stress inducing to the point of suicidality, which was similar to the findings of Czeisler et al. [34] who found the rate of thoughts of suicide within self‐reported unpaid caregivers for adults as being 30.7%. Both these statistics are above the identified rate in 2020–2021 of 1 in 6 (16.7% or around 3.3 million) Australians aged 16–85 reporting serious thoughts about taking their own life at some point in their lives [35]. Mental health family carers' experiences of psychosocial distress and suicidality [36, 37] is a significant and under‐researched area [32, 38]. Active systematic wellbeing screening of mental health family carers is required to address the identified need within this study. Carers need access to timely and appropriate support for extreme distress including thoughts of suicide.

This study confirmed findings in other studies that, especially at the beginning of the pandemic, there was considerably less focus on health impacts and needs of mental health family carers compared to other groups [39] and that there were intersectional vulnerabilities placing undue pressure on mental health family carers [40].

### 4.1 Implications for Practice and Policy

Collaboratively the project identified recommendations arising from the family carer experiences reported in this study. Recommendations for governments and policymakers included (1) to fund the creation of carer peer navigator roles – providing information and support – across inpatient and community services for family carers; (2) to prioritise the creation of carer support on‐call roles, accessible via local and regional mental health triage services, to respond to crises experienced by families; (3) to fund available and responsive mental health carer respite, reducing carer workload so it does not overwhelm family members assisting them to remain in paid work and (4) in recognising the impact of providing long‐term and acute mental health care on the psychosocial wellbeing of carers, establish suicide prevention services responsive to family members experiencing acute and/or cumulative stress and distress. Further, in future pandemics or disaster planning, especially at the beginning of serious incidents, governments, policymakers and mental health services need to consider how to support people with increased psychological distress, alongside reducing uncertainty and managing communications about recommendations and enforceable rules.

### 4.2 Limitations

To provide responsive information to governments, this study was time limited and therefore decisions about recruitment included utilising purposive sampling. This approach also enabled known support for people who may have been more vulnerable due to the COVID‐19 pandemic.

While the aim of the project was to reach a breadth and depth of voices of family carers who support people with mental health challenges, a number of challenges impacted the study. To ensure culturally safe and appropriate support for multicultural family carers, recruitment approaches were undertaken to actively include First Nations and multicultural people through specific reference groups. The time and funding limitations of the study prevented fuller engagement with Aboriginal and Torres Strait Islands people. Two states where responses to the survey were low were experiencing challenges during this time including Queensland with widespread flooding and the Northern Territory were entering their first episode of COVID‐19 presence.

Some families may not have had the technical resources or may have been experiencing high levels of distress or support provision when asked to complete the survey. Fewer responses were received from people newer to providing support. One reason for this could be the survey invitation was administered through national and state family carer organisations and people newer to caring may not be engaged with these organisations. Family carers may have felt too overwhelmed in their caring role to respond. Further longer‐term studies are required to gain understandings from groups not captured within this study.

## 5 Conclusion

The COVID‐19 pandemic increased the level and complexity of support provided by mental health family carers. The closure and restrictions on services led to family, carers and supporters needing to provide extra hours and at times constant support, including maintaining the safety of the service user through monitoring both emotional and physical needs. This study enabled greater depth and understanding of the adverse impacts on family carers' psychological, emotional, social and physical wellbeing, including individual carer and family unit distress. Findings indicated government policy and pandemic responses resulted in family carers fulfilling the role of care provision and support to unwell and extremely distressed people with mental and psychological ill‐health, often without financial, practical or emotional resources. Many families spoke of the challenges of providing support within community and service restrictions and across borders. The impact of these burdens arising through systemic failures led to isolation, feelings of being overwhelmed, physical and emotional distress and reported thoughts of suicide. The COVID‐19 pandemic highlighted prior areas of policy and systemic support that had been missing and also the loss of services that had been of benefit to the service users and family carers.

## Author Contributions

**Caroline Walters:** conceptualisation, investigation, writing–original draft, methodology, validation, visualisation, writing–review and editing, software, funding acquisition, formal analysis, project administration, data curation, resources. **Eileen McDonald:** conceptualisation, investigation, funding acquisition, methodology, visualisation, writing–review and editing, formal analysis. **Carli Sheers:** conceptualisation, investigation, funding acquisition, methodology, visualisation, writing–review and editing, formal analysis. **Kerry Hawkins:** conceptualisation, investigation, funding acquisition, methodology, visualisation, writing–review and editing, formal analysis. **Hayley Solich:** conceptualisation, investigation, funding acquisition, methodology, visualisation, writing–review and editing, formal analysis. **Julie Anne Anderson:** conceptualisation, funding acquisition, methodology, writing–review and editing, project administration. **Nevena Simic:** conceptualisation, investigation, methodology, visualisation, writing–review and editing, formal analysis. **Danielle Moore:** investigation, methodology, visualisation, writing–review and editing, formal analysis. **Tony Stevenson:** investigation, methodology, visualisation, writing–review and editing, formal analysis. **Sharon Lawn:** conceptualisation, investigation, funding acquisition, methodology, visualisation, writing–review and editing, formal analysis. **Melinda Goodyear:** writing–review and editing, methodology, validation, software, formal analysis, supervision, resources, investigation. **Marcelo Maghidman:** investigation, methodology, validation, writing–review and editing, formal analysis, supervision. **Melissa Petrakis:** conceptualisation, investigation, funding acquisition, writing–original draft, methodology, validation, visualisation, writing–review and editing, formal analysis, project administration, data curation, supervision, resources.

## Ethics Statement

The Monash University Human Research Ethics Committee was satisfied the study met the requirements for the National Statement on Ethical Conduct in Human Research and granted approval (Project ID 31516).

## Consent

The front page of the survey had a link to the explanatory statement and people were advised participation in the research was voluntary. Completion of the survey indicated consent to participate.

### 1 Conflicts of Interest

The authors declare no conflicts of interest.

## Data Availability

The data that support the findings of this study are available on request from the corresponding author. The data are not publicly available due to privacy or ethical restrictions. The participants of this study did not give written consent for their data to be shared publicly, so due to the sensitive nature of the research supporting data is not available.
